# The measurement scale of resilience among family caregivers of children with cancer: a psychometric evaluation

**DOI:** 10.1186/s12889-019-7512-8

**Published:** 2019-08-27

**Authors:** Filiberto Toledano-Toledano, José Moral de la Rubia, Yunier Broche-Pérez, Miriam Teresa Domínguez-Guedea, Víctor Granados-García

**Affiliations:** 1 Evidence-Based Medicine Research Unit, Hospital Infantil de México Federico Gómez National Institute of Health, Dr. Márquez 162, Doctores, Cuauhtémoc, 06720 México City, Mexico; 2 Facultad de Psicología, Universidad Autónoma de Nuevo León, Dr. Carlos Canseco, 110, Esq. Dr. Aguirre Pequeño, Col. Mitras Centro, 64460 Monterrey, Mexico; 3 Department of Psychology, Universidad Central “Marta Abreu” de Las Villas, Carretera de Camajuaní Km 5 1/2, 54830 Santa Clara, Villa Clara Santa Cuba; 4 Department of Psychology and Communication Sciences, University of Sonora, Blvd. Luis Encinas y Rosales, Col. Centro S/N, 83000 Hermosillo, Sonora Mexico; 5 Unidad de Investigación Epidemiológica y en Servicios de Salud, Área Envejecimiento, Centro Médico Nacional Siglo XXI, 3er piso. Edificio CORSE, Av. Cuauhtémoc 330. Doctores Cuauhtémoc, 06720 México City, Mexico

**Keywords:** Validity, Reliability, Resilience, Family caregivers, Childhood cancer, Psychometric properties, Social desirability, CFA

## Abstract

**Background:**

Resilience to disease is a process of positive adaptation despite the loss of health, it involves the development of vitality and skills to overcome the negative effects of adversity, risks, and vulnerability caused by disease. In Mexico, cancer is the leading cause of death in children. Both the diagnosis and the treatment of childhood cancer affect the health of family caregivers. However, resilience is a personality trait that can be protective in these situations. Therefore, resilience is an important psychological construct to measure, evaluate and develop in specific populations and contexts. In Mexico, a scale to assess this trait has been developed. This study aimed to test the reliability and factor structure of the Mexican Measurement Scale of Resilience (RESI-M), describe its distribution, evaluate its relationship with sociodemographic variables, and verify its concurrent validity with psychological well-being, depression, anxiety and parental stress and its independence from social desirability.

**Methods:**

A cross-sectional study was conducted involving an intentional nonprobability sample of 330 family caregivers of children with cancer hospitalized at the National Institute of Health in Mexico City. The participants responded to a sociodemographic variables questionnaire, the Mexican Measurement Scale of Resilience RESI-M, and five other assessment scales.

**Results:**

Overall internal consistency was very high (ordinal alpha = .976). The confirmatory factor analysis demonstrated that the five-factor model had a close fit to the data: NFI = .970, CFI = .997, SRMR = .055, and RMSEA = .019. The distributions of the RESI-M total score followed a normal distribution. The RESI-M total score correlated positively with psychological well-being and negatively with depression, parental stress and anxiety. The overall RESI-M total score also correlated positively with age, but there was no difference in means between women and men. Resilience was independent of social desirability.

**Conclusions:**

The RESI-M shows reliability and construct validity in family caregivers of children with cancer and does not show a bias in relation to social desirability.

## Background

In the US, cancer is the second leading cause of death after accidents in the infant population aged 5 to 14. Ten thousand cases of infant cancer were diagnosed in 2017, representing 1% of diagnosed cancer cases [1]. The types of cancer common in infant populations are acute lymphoblastic leukemia (26%), brain and central nervous system tumors (21%), neuroblastoma (7%) and non-Hodgkin lymphoma (6%) [2]. The prevalence of cancer in the US infant population is higher in white and Hispanic ethnic groups. Due to advances in treatment, the 5-year survival rate for children diagnosed with cancer is greater than 80% [2].

In Mexico, child cancer is a public health problem and is the leading cause of death in children aged 5 to 14. Five thousand cases of childhood cancer are diagnosed annually in Mexico, representing 5% of diagnosed cancer cases [3]. The 5-year survival rate of children diagnosed with cancer in Mexico is 56%. Approximately 75% of cancer diagnoses in Mexico are performed in advanced stages of the disease [3]. The most common types of childhood cancer in Mexico are acute lymphoblastic leukemia, acute myeloblastic leukemia, non-Hodgkin lymphoma and Hodgkin disease [3].

Children with a diagnosis of cancer represent one of the greatest challenges for family environments, with physical, psychological, socioeconomic, and behavioral effects on patients and their caregivers. These effects translate into vulnerability and a decline in families’ quality of life and functioning [4]. However, despite adversity, families and caregivers of pediatric patients can adapt to the diagnosis and medical treatment crises [5]. Families that adapt are proactive, gather information, find resources, form cooperative and support networks with medical personnel, and establish social relations [6].

In this context, resilience plays a central role in addressing and overcoming disease. Because there are psychosocial factors related to caregiver burden among families of children with chronic conditions, including sociocultural historical premises, parental stress, anxiety, social support networks, family support, family functioning, well-being and sociodemographic characteristics, these variables influence the processes of resilience of families facing adversity, risk and vulnerability during a child’s disease [7].

Family-focused studies on resilience help in understanding the risks and protective processes involved in attaining positive development in contexts of adversity [8]. Research shows that especially when parents are motivated to change, cooperate with health professionals, and communicate the effects of treatment to their children, a favorable prognosis for the disease is fostered and idiosyncrasies in the family’s progression and resilience are reduced [9].

While measurement constructs related to resilience are of paramount methodological importance, several authors highlight specific challenges and shortcomings in this area. For example, Windle et al. [10] conducted a systematic review of 19 scales focused on the resilience resources of individuals and found that most of these scales lacked information regarding psychometric properties and required additional validation. At the time, the best-rated scales included the Connor-Davidson Scale, the Resilience Scale for Adults, and the Brief Resilience Scale. Additionally, the need for effective instruments that assess strengths, protective processes, and outcomes based on these resilience resources in the context of pediatric disease is well acknowledged [11].

Empirical findings have been reported based on assessment instruments created mainly in Europe and the United States, but extrapolating the results of their implementation to other contexts and cultures is challenging [10]. In response to this situation, Palomar and Gómez created the Mexican Scale of Resilience (RESI-M) [12]. The RESI-M is based on the Connor-Davidson Resilience Scale [13] and the Resilience Scale for Adults [14].

From the combination of these two scales, Mexican researchers defined five factors based on a principal component analysis with a Varimax rotation and determined the number of factors through the Kaiser criterion. The first factor of personal competence is characterized by the conviction that one is sufficiently prepared to be able to face any situation that arises, even if it is unexpected. The second factor comprises the features of self-confidence, tolerance to negative situations, and the ability to strengthen oneself when faced with the effects of stress. The third factor concerns secure relationships and acceptance of change, or the ability to establish personal relationships of support and personal development as well as the ability to flexibly adapt to new situations. The fourth factor, named control, is characterized by the ability to promote one’s well-being and to conduct oneself according to what one wants or plans. The five factors of spiritual influence are characterized by the positive effect that spiritual beliefs and practices have on the person.

Resilience to disease is a process of positive adaptation despite the loss of health, it involves the development of vitality and skills to overcome the negative effects of adversity, risks, and vulnerability caused by disease [15]. The existence of a scale in Spanish that enables the measurement of resources for resilience to cancer in childhood and that has had sufficient analysis of its psychometric properties may extend the research conducted with family caregivers of children with chronic disease. In addition, this assessment instrument can contribute significantly to the development and evaluation of intervention programs aimed at families to help overcome adversity in the face of disease.

In a sample of 446 Mexican family caregivers of children with different chronic diseases, the factor validity and internal consistency of the RESI-M was studied [15]. The expected five-factor model showed a close fit to the data through maximum likelihood estimation, χ^2^/df = 1.66, CFI = .95, and RMSEA = .03 (90% CI: .02, .04). The internal consistency for each factor using Cronbach’s alpha ranged from .76 to .93, and the overall internal consistency was .95. No average difference in the RESI-M and its factors was found between women and men [15]. This validation study did not use methods designed for ordinal variables (Likert-type items), such as the ordinal alpha, polychoric correlation matrix, and free-scale least squares methods. The distribution and convergent/divergent validity of the RESI-M were not studied.

The RESI-M has also been validated in a sample of 120 Mexican women with cancer [16]. The internal consistency value for the scale was very high, Cronbach’s alpha = .96, and the internal consistency the factors ranged from very high, .93, to high, .82. The 5-factor model showed a close fit to the data when items 2 and 15 were eliminated. Fit indexes through unweighted least squares estimation were Bollen-Stine bootstrap probability = .072, GFI = .968, AGFI = .963, NFI = .960, and RFI = .957 [16]. The distribution of the scale or the factors did not adjust to a normal curve. In this study, neither the ordinal alpha nor the polychoric correlation matrix was used.

In a sample of 348 healthy Mexican adults (235 women and 113 men), the factorial weights pattern was reproduced through component analysis with 43.60% of the variance of the indicators explained by the five factors. The overall internal consistency was very high (Cronbach’s alpha = .92), and the internal consistency values for the factors were high (.86 to .83), except for the Structure factor, which had low internal consistency (Cronbach’s alpha = −.59) [17].

A negative correlation between depression and anxiety and stress and resilience has been reported using the RESI-M [18], but its relationship with social desirability has not been studied. Social desirability is a potential bias that can be present in the evaluation of traits [19].

Given the need for an assessment instrument to measure resilience among family caregivers of children with cancer and the methodological background of validation studies with the RESI-M in Mexico, the objectives of this research are as follows: 1) calculate the internal consistency through ordinal alpha; 2) contrast factorial construct validity, verifying the convergent and discriminant validity of five RESI-M factors, from polychoric matrix through free-scale least squares estimation; 3) describe the distribution of scores in the RESI-M and its five factors; 4) compare the means between the factors; 5) evaluate the relation of the RESI-M total score and its factors with the sociodemographic variables of educational level, age and sex; and 6) test the convergent validity with respect to psychological well-being, the divergent validity with respect to depression, anxiety and parental stress, and the independence in relation to social desirability.

In correspondence with the proposed objectives, the hypotheses are as follows: 1) very high overall internal consistency [12, 15–17] and from very high to acceptable internal consistency for the factors [12, 15, 16]; 2) a five-factor model with convergent and discriminant validity in its factors (strength/self-confidence, social competence, family support, social support, and structure) [12, 15–17]; 3) normal distribution in the RESI-M total score due to assessing a personality trait [20], although its factors may show asymmetry [16, 17]; 4) the higher the level of resilience, the higher the educational level and age [12, 21, 22], and greater resilience in women than men [12, 22–24], although sociodemographic variables are usually independent of resilience [15, 16, 24]; 5) the highest means in family and social support and the lowest mean in structure [16], and 6) positive correlation with psychological well-being [14], negative correlation with depression, anxiety and parental stress [18], and independence or low correlation with social desirability.

## Methods

### Participants

A total of 330 family caregivers of hospitalized children with cancer were interviewed at the Hospital Infantil de México Federico Gómez National Institute of Health, in Mexico City. This instrumental-type empirical study was conducted using a cross-sectional nonexperimental design with intentional nonprobability sampling. This hospital receives approximately 320 new cases of children under 18 years of age with some type of cancer annually [25]. Therefore, the case rate was greater than 90% of the annual incidence of cancer in the hospital.

The inclusion criteria used for this study were as follows: 1) older than 18 years of age, 2) the father or mother or the family caregiver of a child hospitalized due to cancer at the Hospital Infantil de México Federico Gómez National Institute of Health, and 3) having read and signed an informed consent form.

### Procedure

Data collection was performed by trained personnel in the Evidence Based Medicine Research Unit of the Hospital Infantil de México Federico Gómez National Institute of Health under the direction of the first author of this article. The data collection process lasted approximately 4 months of July to October 2018. Family caregivers were contacted in the hospitalization rooms of the National Institute of Health, where their children received treatment. They were then asked to participate in the study. The objectives of the study were explained, and any doubts they had were clarified. Caregivers who agreed to participate signed the informed consent and subsequently answered the following self-report instruments, which were administered individually and in a single session. After delivering the questionnaire, the interviewer left the room. Approximately 1 hour later, the interviewer returned to resolve any questions the participants had and to collect the self-report instruments.

### Ethical considerations

The protocol of the present study was approved by the Ethics and Biosafety Committee of the Hospital Infantil de México Federico Gómez National Institute of Health. All participants were provided with information regarding the study’s objective and their research rights, particularly regarding the fact that there were no consequences if they decided not to participate. Parents who decided to participate were provided with instructions on how to answer the questionnaire, and they completed the questionnaires by themselves in the room where their child was hospitalized. Participation in this study was voluntary. Prior to completion, participants were all informed of their rights as outlined by the Helsinki Declaration [26]. This study also adhered to the ethical rules and considerations for research with humans currently in force in Mexico [27] and those developed by the American Psychological Association [28].

### Measurement instrument

#### A sociodemographic variables questionnaire for research on family caregivers of children with chronic diseases (Q-SV)

This instrument comprises 20 items that measure individual, familial, and caregiver factors such as age, gender, and marital status, among others. In addition, this instrument includes the child’s sex, age, diagnosis, and length of hospitalization.

#### Mexican resilience scale (RESI-M)

The original scale consisted of 43 items on a 4-point Likert-type scale (1 = “totally disagree” to 4 = “totally agree”). It was created by Palomar and Gómez [12] and measures the level of global resilience. In an incidental sample of 217 Mexican participants from the general population, the overall internal consistency was very high (Cronbach’s alpha = .93). It has a five-factor structure: strength and self-confidence (19 items, Cronbach’s alpha = .93), social competence (8 items, Cronbach’s alpha = 0.87), family support (6 items, Cronbach’s alpha = .87), social support (5 items, Cronbach’s alpha = .84), and structure (5 items, Cronbach’s alpha = .79) [12]. Scores on the scale and its five factors are obtained by adding the scores of each item. Higher scores indicate higher levels of resilience. See Appendix.

#### Beck depression inventory, second edition (BDI-II)

The BDI-II has been validated in the population of family caregivers of children with chronic diseases [29]. This self-report instrument consists of 21 items to measure symptoms of depression. Participants responded using a four-point scale (0 to 3), with higher scores indicating greater depressive symptomology. The BDI-II is composed of two factors: “depressed mood and motor complaints” and “negative cognitions”. Its overall internal consistency was very high in a student sample, Cronbach’s alpha = .91, and high in a community sample, Cronbach’s alpha = .87 [29].

#### Beck anxiety inventory (BAI)

The BAI has been validated in a Mexican population [30]. This instrument consists of 21 items that measure anxiety. There are four response choices (0 = “little or none” to 3 = “severely”), with total scores ranging from 0 to 63. Higher scores indicate greater levels of anxiety. The BAI is composed of two factors: somatic and cognitive symptoms. Its overall internal consistency is high, Cronbach’s alpha = .83 [30].

#### Parental stress scale (PSS)

The version of the PSS used for this study was based on the Spanish adaptation by Oronoz et al. [31]. The scale comprises 12 items with five Likert-type response options (1 = “totally disagree” to 5 = “totally agree”) and has two factors with adequate values of internal consistency (rewards, Cronbach’s alpha = .77, and stressors. Cronbach’s alpha = .76) [31]. Higher scores indicate greater parental stress.

#### Psychological well-being scale (PWS)

The linguistic adaptation of the PWS for the current study, using the translation-retranslation strategy, was based on the instrument from Bech et al. [32], which contains 10 items with four Likert-type response options (0 = “never” to 3 = “all the time”). A higher score indicates greater psychological well-being. Its overall internal consistency is very high (Cronbach’s alpha = .90) [32].

#### Marlowe-Crowne social desirability scale (MCSC)

This self-report instrument consists of 33 items in which respondents are asked to choose true or false answers. Its scores range from 0 to 33. High scores on this scale show a tendency to present oneself in a socially acceptable manner that conforms to others’ expectations. Scores between 0 to 8, 9 to 19, and 20 to 33 indicate low, moderate, and high levels of social desirability, respectively. It was translated into Spanish [33]. Among Spanish university students, its overall internal consistency was acceptable (Cronbach’s alpha = .78), with a mean of 15.83 and a standard deviation of 5.15 [33].

### Statistical analyses

For the analysis data, a confirmatory factor analysis (CFA) was performed. Parameters and goodness-of-fit indices were estimated using the Scale-Free Least Squares (SLS) method. Standard errors were calculated by the percentile bootstrap method with the extraction of 2000 samples. Seven goodness-of-fit indices were assessed: relative chi-square (χ^2^/df), Standardized Root Mean Square Residual (SRMR), Jöreskog and Sörbom’s Goodness-of-Fit Index (GFI) and Adjusted Goodness-of-Fit Index (AGFI), Bentler and Bonett’s Normed Fit Index (NFI), Bentler’s Comparative Fit Index (CFI), and Bollen’s Relative Fit Index (RFI). The criteria to establish that the proposed models showed a close fit to the data were χ^2^/df ≤ 2, SRMR ≤ .08, AGFI ≥ .90, and GFI, NFI, CFI, and RFI ≥ .95 [34]. The parsimony of the model was tested using the James-Mulaik-Brett parsimony ratio (PR ≥ .80 high) and the parsimonious indexes for GFI (PGFI ≥ .50 adequate, and ≥ .70 high), NFI and CFI (PNFI and PCFI ≥ .60 adequate, and ≥ .80 high) [34].

After applying CFA, the convergent validity of each factor and the discriminant validity between pairs of factors were examined. An average variance extracted (AVE) greater than .50 and a composite reliability (McDonald’s omega coefficient) greater than .70 were considered evidence of convergent validity. A shared correlation between two factors, both lower than .70 and lower than the AVE from each factor, was used as a criterion for discriminant validity [35].

The internal consistency was calculated through the ordinal alpha. A value of ordinal alpha ≥ .70 was interpreted as reflecting acceptable internal consistency, ≥ .80 indicated high internal consistency, and ≥ .90 indicated very high internal consistency [36].

The distributions of scores in the RESI-M and its five factors were described through measures of central tendency (arithmetic mean and median), variation (standard deviation), shape (moment coefficients of skewness and excess kurtosis for sample), and moments (percentile). The null hypothesis of normal distribution was tested using the Pearson-Agostino omnibus test [37].

Comparison among factor means was performed using a one-way repeated-measures analysis of variance (ANOVA), and posterior comparisons between the pairs of factors were tested using Student’s t-test for paired samples with the Bonferroni correction for the significance level.

The relationships between the RESI-M and sociodemographic variables were examined through biserial-point correlation coefficient with sex, Spearman rank correlation coefficient with educational level, and Pearson product-moment correlation coefficient with age. To examine concurrent validity and determine the bias introduced by social desirability, the Pearson product-moment correlation coefficient (r) was calculated. Absolute values of the correlation coefficients lower than .10 were interpreted as indicating a trivial strength of association, from .10 to .299 indicated a low strength of association, from .30 to .499 indicated medium strength, from 50 to .699 indicated high strength, from .70 to .899 indicated very high strength, and higher than or equal to .90 were unitary [38]. Confidence intervals and levels of significance were calculated using the Efron bootstrap percentile method with 1000 replications, except for assessment scale scores with normal distribution. Statistical calculations were performed with the SPSS v.24, IBM Inc., Chicago, USA, AMOS (version 16), and Excel 2007.

## Results

### Sociodemographic characteristics of participants

The sample comprised 272 (82%) women and 58 (17.6%) men. Participants ranged in age from 18 to 63 years, with an average age of 32.60 (SD = 8.59). Regarding education, 3.3% of the participants had no education, 18.5% had primary school education, 46.7% had secondary school education, 24.8% had upper secondary (high school) education, and 6.7% had university or college education. The median and mode of the number of children was two, ranging from 0 to 10. For more details, see Table 1.

**Table 1:** Summary statistics of sociodemographic variables

Sociodemographic variable		n	%	M (95% CI)	Mdn
Sex	Women	272	82.4		
	Men	58	17.6		
Schooling	No schooling	11	3.3		
	Primary	61	18.5		
	Secondary	154	46.7		
	Higher secondary (high school)	82	24.8		
	University or college	22	6.7		
Occupation	Housewife	221	67.0		
	White-collar worker	41	12.4		
	Merchant	30	9.1		
	Unemployed	23	7.0		
	Blue-collar worker	11	3.3		
	Student	4	1.2		
Marital status	Married	138	41.8		
	Living together	115	34.8		
	Separated	31	9.4		
	Single mothers	26	7.9		
	Divorced	10	3.0		
	Widowed	5	1.5		
	Other	5	1.5		
Income per month	< 141 US dollars	201	61		
	Between 141 and 281 US dollars	71	21.5		
	Between 282 and 563 US dollars	49	14.8		
	≥ 564 US dollars	9	1.2		
Religious adscription	Catholic Christian	270	81.8		
	Non-Catholic Christian	36	10.9		
	No religion	24	7.3		
Age (years)				32.602 (31.672, 33.534)	32
Number of children				2.394 (2.258, 2.530)	2

N *=* simple absolute frequency, % = simple percentage, M = arithmetic mean (95% confidence interval), Mdn = Median

Of the pediatric patients cared for by the caregivers, approximately half were female (52%) and approximately half were male (48%). The pediatric patients ranged in age from 1 to 17 years, with an average age of 6.33 (SD = 5.13). In most cases, the elapsed time since the diagnosis of cancer was between 1 week and 1 year (68.5%), and the time since hospitalization was from 1 week to 1 month (83.9%).

### CFA, internal consistency, convergent and divergent validity of factors

**Fig. 1:**
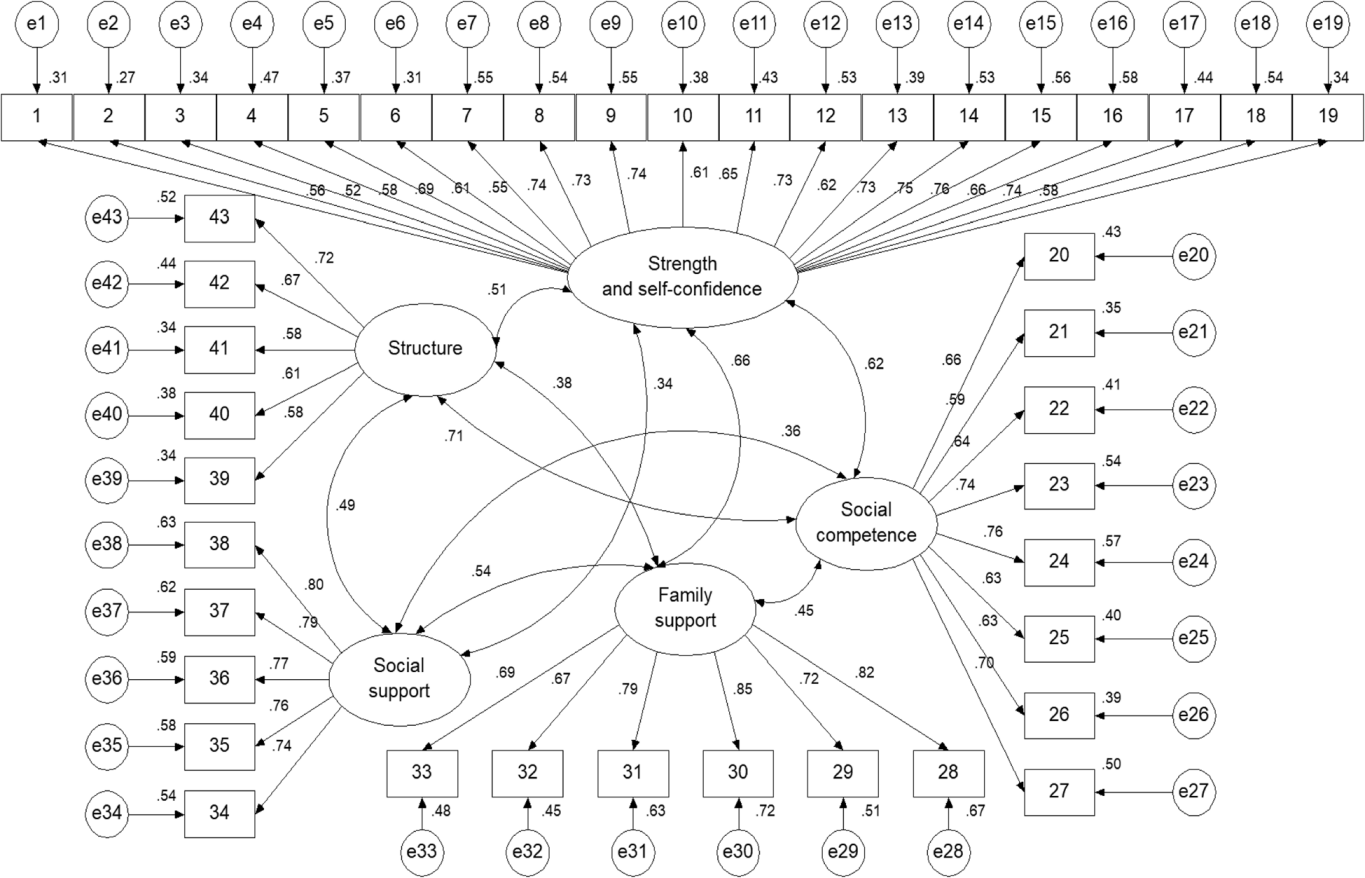
Five-factor model estimated through scale-free least squares

A model of five correlated factors was specified (Fig. 1). All parameters of the model were significant (43 standardized regression weights, 15 correlations and 48 variances) because the bounds of their 95% confidence intervals had the same sign, not including zero (Table 2).

**Table 2:** Parameter estimates with a 95% confidence interval

λ		S_e_^2^		S_F_^2^		r	
F1 → item1	.556 (.460, .644)	S^2^_e1_	.353 (.274, .443)	S_F1_^2^	.158	r_F1,F2_	.624
F1 → item2	.516 (.418, .602)	S^2^_e2_	.381 (.302, .459)		(.105, .219)		(.527, .716)
F1 → item3	.585 (.485, .671)	S^2^_e3_	.240 (.193, .293)			r_F1,F3_	.656
F1 → item4	.689 .615, .754)	S^2^_e4_	.231 (.185, .281)				(.567, .737)
F1 → item5	.607 (.501, .695)	S^2^_e5_	.307 (.237, .387)			r_F1,F4_	.339
F1 → item6	.554 (.444, .650)	S^2^_e6_	.351 (.276, .437)				(.205, .466)
F1 → item7	.739 (.678, .792)	S^2^_e7_	.139 (.112, .166)			r_F1,F5_	.512
F1 → item8	.732 (.677, .783)	S^2^_e8_	.164 (.130, .197)				(.390, .624)
F1 → item9	.743 (.686, .793)	S^2^_e9_	.132 (.104, .161)				
F1 → item10	.613 (.539, .686)	S^2^_e10_	.245 (.188, .310)				
F1 → item11	.653 (.554, .738)	S^2^_e11_	.246 (.180, .320)				
F1 → item12	.729 (.652, .790)	S^2^_e12_	.190 (.145, .240)				
F1 → item13	.624 (.553, .689)	S^2^_e13_	.221 (.173, .274)				
F1 → item14	.726 (.657, .786)	S^2^_e14_	.233 (.182, .284)				
F1 → item15	.750 (.689. .806)	S^2^_e15_	.142 (.112, .171)				
F1 → item16	.761 (.702, .816)	S^2^_e16_	.182 (.142, .223)				
F1 → item17	.660 (.573, .736)	S^2^_e17_	.256 (.195, .323)				
F1 → item18	.737 (.666, .799)	S^2^_e18_	.168 (.133, .205)				
F1 → item19	.583 (.476, .677)	S^2^_e19_	.226 (.172, .290)				
F2 → item20	.659 (.547, .746)	S^2^_e20_	.286 (.223, .355)	S_F2_^2^	.220	r_F2,F3_	.447
F2 → item21	.589 (.457, .700)	S^2^_e21_	.353 (.262, .447)		(.138, .305)		(.334, .558)
F2 → item22	.638 (.536, .727)	S^2^_e22_	.327 (.252, .405)			r_F2,F4_	.365
F2 → item23	.735 (.647, .810)	S^2^_e23_	.241 (.175, .315)				(.225, .497)
F2 → item24	.758 (.677, .829)	S^2^_e24_	.231 (.171, .293)			r_F2,F5_	.706
F2 → item25	.630 (.540, .717)	S^2^_e25_	.305 (.240, .368)				(.591, .796)
F2 → item26	.626 (.517, .716)	S^2^_e26_	.250 (.195, .313)				
F2 → item27	.704 (.603, .792)	S^2^_e27_	.229 (.165, .295)				
F3 → item28	.819 (.731, .892)	S^2^_e28_	.155 (.091, .229)	S_F3_^2^	.189	r_F3,F4_	.536
F3 → item29	.717 (.611, .814)	S^2^_e29_	.178 (.121, .241)		(.131, .261)		(.400, .660)
F3 → item30	.849 (.791 .901)	S^2^_e30_	.133 (.087, .183)			r_F3,F5_	.381
F3 → item31	.791 (.721, .854)	S^2^_e31_	.189 (.134, .245)				(.249, .508)
F3 → item32	.673 (.560, .773)	S^2^_e32_	.253 (.172, .335)				
F3 → item33	.690 (.585, .784)	S^2^_e33_	.194 (.140, .252)				
F4 → item34	.737 (.634, .835)	S^2^_e34_	.207 (.127, .289)	S_F4_^2^	.246	r_F4,F5_	.489
F4 → item35	.761 (.668, .843)	S^2^_e35_	.206 (.130, .292)		(.173, .333)		(.368, .602)
F4 → item36	.766 (.653, .859)	S^2^_e36_	.224 (.133, .321)				
F4 → item37	.789 (.684, .879)	S^2^_e37_	.169 (.098, .250)				
F4 → item38	.796 (.677, .895)	S^2^_e38_	.164 (.087, .248)				
F5 → item39	.584 (.466, .684)	S^2^_e39_	.356 (.275, .440)	S_F5_^2^	.184		
F5 → item40	.615 (.497, .718)	S^2^_e40_	.327 (.248, .406)		(.112, .262)		
F5 → item41	.584 (.474, .678)	S^2^_e41_	.298 (.225, .372)				
F5 → item42	.666 (.549, .764)	S^2^_e42_	.210 (.149, .280)				
F5 → item43	.718 (.623, .803)	S^2^_e43_	.274 (.202, .348)				

Method to minimize the discrepancy function between the empirical covariance matrix and a covariance matrix implied by the model: free-scale least squares, bootstrap method to estimate confidence intervals: percentile. λ = standardized measurement weight, S_e_^2^ = residual variance, S_F_^2^ = factor variance, r = correlation between factors through Pearson product-moment correlation coefficient. Factors: F1 = strength and self-confidence, F2 = social competence, F3 = family support, F4 = social support, F5 = structure. Source: Prepared by the authors

The overall AVE was close to .50 (AVE = .475), and the overall composite reliability was very high (ω = .975). Overall internal consistency was also very high (ordinal α = .976). The factors of family support and social support showed convergent validity (AVE = .577 and ω = .890 for the former and AVE = .593 and ω = .879 for the latter) and internal consistency (ordinal alpha = .930 and .924, respectively). The factors of strength/self-confidence, social competence and structure showed internal consistency (ordinal alpha = .956, .900, and .828, respectively). Although their AVE values were lower than .50, they were higher than .40 (AVE = .443, .448 and .404, respectively), and their McDonald’s omega coefficients were higher than .70 (ω = .937, .866 and .771, respectively); therefore, these three factors had acceptable convergent validity (Table 3).

**Table 3:** Internal consistency, convergent validity, summary statistics, and normality test

Statistics	Mexican Resilience Scale (RESI-M)					
	Total score	Strength and self-confidence	Social competence	Family support	Social support	Structure
Internal consistency and convergent validity						
NI	43	19	8	6	5	5
Ordinal α	.976	.956	.900	.930	.924	.828
α	.949	.936	.867	.888	.879	.773
AVE	.475	.443	.448	.577	.593	.404
ω	.975	.937	866	.890	.879	.771
Summary statistics						
[Min, Max]	[1.93, 3.98]	[2.05, 4]	[1.25, 4]	[1, 4]	[1, 4]	[1, 4]
M (95% CI)	3.086 (3.046, 3.127)	3.150 (3.103, 3.197)	2.847 (2.792, 2.903)	3.287 (3.230, 3.345)	3.208 (3.147, 3.269)	2.864 (2.809, 2.919)
Mo	3	3	3	3	3	3
SD	0.375	0.433	0.513	0.534	0.566	0.508
G_1_	0.233 (−0.030, 0.496)	0.238 (−0.025, 0.501	0.201 (−0.062, 0.464)	−0.682 (− 0.945, − 0.419)	−0.496 (− 0.759, − 0.233)	0.160 (− 0.103, 0.423)
G_2_	0.106 (− 0.419, 0.631)	− 0.555 (−1.080, − 0.030)	0.332 (− 0.193, 0.857)	0.861 (0.336, 1.386)	0.598 (0.073, 1.123)	0.698 (0.173, 1.223)
P10	2.677	2.632	2.25	2.683	2.6	2.2
P20	2.791	2.842	2.375	3	3	2.4
P25	2.84	2.895	2.5	3	3	2.6
P30	2.907	2.895	2.625	3	3	2.6
P40	2.954	3	2.750	3	3	2.8
P50	3.023	3.053	2.875	3.333	3	2.8
P60	3.116	3.158	3	3.5	3	3
P70	3.279	3.368	3	3.667	3	3
P75	3.331	3.474	3	3.667	3	3
P80	3.395	3.526	3.125	3.833	3	3.2
P90	3.626	3.842	3.625	4	4	3.6
Goodness-of-fit test for a normal distribution						
PA	3.180^ns^	7.443^*^	3.785^ns^	36.225^***^	18.680^***^	8.209^*^

Sample size: *N* = 330. Internal consistency: NI = number of items, Ordinal α = ordinal coefficient alpha, and α = Cronbach’s alpha based on standardized items. Convergent validity: *AVE* average variance extracted, ω = McDonald’s omega coefficient. Summary statistics: [Min, Max] = observed range or interval between minimum and maximum value (potential range from 1 to 4), M = arithmetic mean (95% confidence interval using t-distribution), *Mo* mode, *SD* standard deviation, G_1_ = Fisher-Pearson skewness coefficient for sample (95% confidence interval using z-distribution; standard error: G_1_SE = 0.134), G_2_ = Fisher-Pearson excess kurtosis coefficient for sample (95% confidence interval using z-distribution; standard error: G_2_SE = 0.268), Pn = nth percentile. Normality: PA = Pearson-Agostino omnibus test for a null hypothesis that the data are normally distributed: PA = (G_1_/G_1_SE)^2^ + (G_2_/G_2_SE)^2^; levels of significance for one-tailed test: non-significant (ns) *p* > .05 * *p* ≤ .05, ** *p* ≤ .01, *** *p* ≤ .001. Source: Prepared by the authors

Shared variances between factors ranged from .115 to .498 with an average of .270. Nine of the 10 squared correlations between the five factors were lower than the AVEs corresponding to each pair of correlated factors. Only the shared variance between social competence and structure (*r*^*2*^ = .498) was higher than the AVEs of both factors (.448 and .404, respectively), but this squared correlation was lower than .50; therefore, 10 factors showed discriminant validity.

The five-factor model fit to the data was close through Free-scale Least Squares estimation (χ^2^ = 956.331, df = 850, χ^2^/df = 1.125, GFI = 975, AGFI = .973, NFI = .970, RFI = .968, CFI = .997, SRMR = .055, and RMSEA = .019). Additionally, the parsimony of the model was high (PR = .941, PGFI = .876, PNFI = .913, and PCFI = .938).

### Distribution of the RESI-M total score and its factors

The distributions of the total score (PA =3.180, *p* = .204, and JB =3.061, *p* = .216) and the social competence factor (PA =3.785, *p* = .151, and JB =3.519, *p* = .172) followed a normal distribution. Scores for the strength and self-confidence factor (PA = 7.443, *p* = .024) and the structure factor (PA = 8.209, *p* = .017) approached a normal distribution. In both cases, the null hypothesis of normal distribution can be maintained at a significance level of 0.01, and the coefficients of skewness and excess kurtosis can be considered null. The scores in the family and social support factor showed bias toward values below the median and had sharp profiles or weighted tails (Table 3).

### Comparison of means between the five factors

**Fig. 2:**
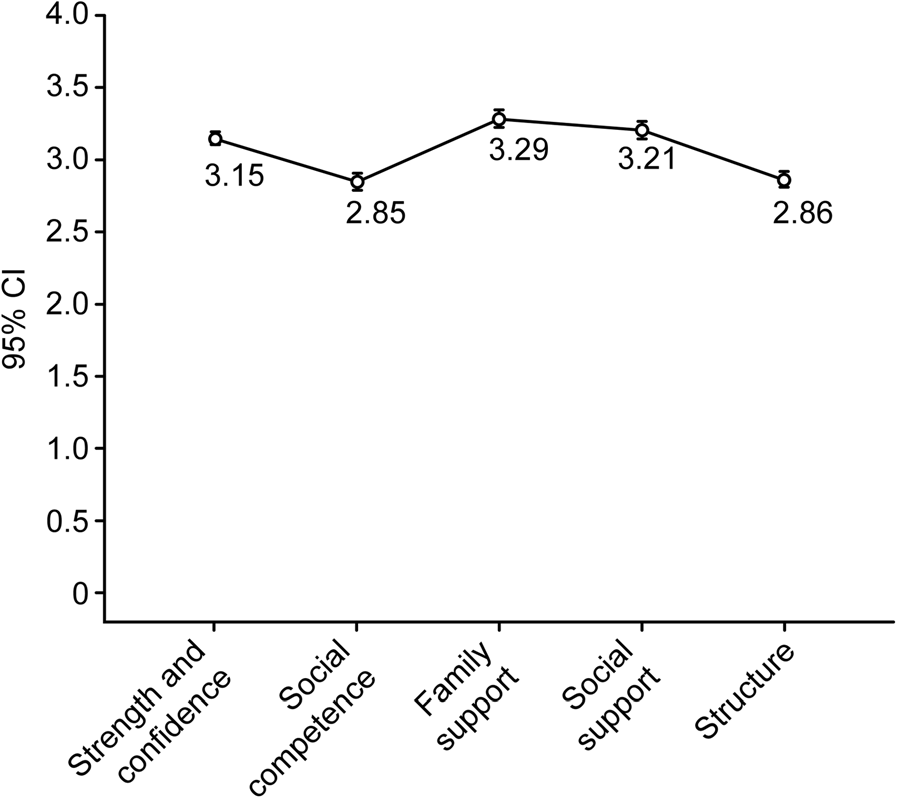
Mean plot with standard error bars of the RESI-M five factors

When comparing mean scores on the five factors through the repeated-measures ANOVA, there was a significant difference (Greenhouse-Geisser correction for the test of within-subjects effects: F[4, 384] = 90.497, *p* < .001, without assuming sphericity due to the rejection of the null hypothesis that the error covariance matrix of the dependent variables with an orthonormal transformation is proportional to an identity matrix through the Mauchly test: W = 0.668, χ^2^[9] = 132.056, *p* < .001. Thus, a correction factor for degrees of freedom was used: ε = .846). The size of the effect of the five factors on the underlying dimension (resilience) was large (φ^2^ = .216). The highest means corresponded to the factors of family support, social support and strength/self-confidence, and the lowest ones corresponded to the factors of structure and social competence (Fig. 2). Posterior comparisons were performed using Student’s t-test for paired samples. The Bonferroni correction was applied to the significance level (2*α /[k*(k-1)] = 0.1/20 = .005). With this significance level, seven of the 10 comparisons were significant, and there were no differences in three comparisons. The mean of social support was statistically equivalent to the means of strength/self-confidence (t[329] = − 1.767, *p* = .078) and family support (*t*[329] = 2.568, *p* = .011). Additionally, the means of social competence and structure were statistically equivalent (t[329] = − 0.650, *p* = .516).

### Relationship with sociodemographic variables

The RESI-M total score and its factors of social competence, family support and social support were positively correlated with educational level with a low strength of association. The other two factors were independent of educational level. The correlation between structure and age was significant and positive and had a low strength of association, but the total score and the remaining factors were independent of age. The RESI-M total score and its five factors were independent of sex (Table 4).

**Table 4:** Correlation with sociodemographic variables and scales of assessment

Var.	CC	Mexican Resilience Scale (RESI-M)					
		Total score	Strength self-conf.	Social compet.	Family support	Social support	Structure
Sociodemographic variables							
Sex	r_bp_ (BS)	.01^ns^ (−.09, .12)	.04^ns^ (−.06, .14)	-.04^ns^ (−.14, .07)	.05^ns^ (−.04, .14)	-.04^ns^ (−.14, .07)	.01^ns^ (−.10, .11)
Educ. Level	r_S_ (BS)	.141^**^ (.03, .24)	.080^ns^ (−.03, .18)	.145^***^ (.03, .25)	.115^*^ (.02, .22)	.187^***^ (.09, .28)	.025^ns^ (−.09, .13)
Age	r (BS)	.07^ns^ (−.05, .19)	.11^ns^ (−.01, .21)	.03^ns^ (−.09, .15)	-.01^ns^ (−.11, .09)	-.08^ns^ (−.18, .02)	.14^**^ (.03, .26)
Scales of assessment							
BDI	r (BS)	−.49^***^ (−.58, −.40)	−.49^***^ (−.56, −.41)	−.34^***^ (−.43, −.24)	−.45^***^ (−.55, −.33)	−.19^***^ (−.30, −.08)	−.23^***^ (−.34, −.12)
BAI	r (BS)	−.28^***^ (−.38, −.18)	−.27^***^ (−.38, −.16)	−.22^***^ (−.31, −.12)	−.27^***^ (−.38, −.14)	-.10^ns^ (−.21, .01)	−.13^*^ (−.23, −.03)
PSS	r (BS)	−.35^***^ (−.43, −.25)	−.33^***^ (−.42, −.24)	−.23^***^ (−.31, −.09)	−.32^***^ (−.42, −.22)	−.23^***^ (−.33, −.11)	−.13^*^ (−.23, −.03)
PWS	r	.50^***^ (.41, .57)^FZ^	.50^***^ (.42, .58) ^FZ^	0.32^***^ (.22, .41)^FZ^	0.38^***^ (.27, .47)^BS^	0.24^***^ (.12, .36)^BS^	.27^***^ (.17, .37)^FZ^
MCSDS	r	.10^ns^ (−.02, .20)^FZ^	.07^ns^ (−.04, .18)^FZ^	.07^ns^ (−.03, .18)^FZ^	.12^*^ (.01, .21)^BS^	.07^ns^ (−.03, .16)^BS^	.05^ns^ (−.06, .16)^FZ^

Variables (Var.): Sex (0 = female and 1 = male), Educational level (0 = illiterate to 5 = postgraduate), *BDI* Beck Depression Inventory-II, *BAI* Beck Anxiety Inventory, *PSS* Parental Stress Scale, *PWS* Psychological Well-being Scale, *MCSDS* Marlowe-Crowne Social Desirability Scale. Correlation coefficients (CC): r_bp_ = Point-biserial correlation coefficient, r_S_ = Spearman rank correlation coefficient, r = Pearson product-moment correlation coefficient. BS = Confidence intervals and levels of significance through Efron bootstrap percentile method with 1000 replications. FZ = Intervals and levels of significance for a two-tailed test using Fisher Z-transformation. Levels of significance: non-significant (ns) *p* > .05 *p ≤ .05, ** *p* ≤ .01, *** *p* ≤ .001. Source: Prepared by the authors

### Concurrent validity and relationship with social desirability

The total scores of the scales of psychological well-being and social desirability followed a normal distribution when the null hypothesis of normality was tested through the Pearson-Agostino omnibus test. The confidence intervals of Pearson product-moment correlation coefficients were calculated through Fisher’s Z transformation. However, the total scores of the scales of depression, anxiety and parental stress did not follow a normal distribution (Table 5). In these three latter cases, intervals of Pearson product-moment correlation coefficients and their significance values (*p*-value) were calculated using Efron’s bootstrap percentile method.

**Table 5:** Ranges, measures of central tendency, and normality test

Var.	NI	[Min, Max]	M (95% CI)	Mdn	Mo	SD	α	Ordinal α	PA
Age		[18, 63]^e^	32.603 (31.672,33.534)	32	31	8.593			8.977^*^
BDI	21	[0, 63]^p^ [0, 55]^e^	13.952 (12.875, 15.028)	13	14	9.940	.906	.941	41.958^***^
BAI	21	[0. 63]^p^ [0, 63]^e^	14.115 (12.775, 15.456)	10	6	12.380	.933	.954	96.156^***^
PSS	12	[12, 60]^p^ [12, 40]^e^	19.739 (18.977, 20.502)	18	12	7.039	.853	.919	41.253^***^
PWS	10	[0, 30]^p^ [3, 30]^e^	18.094 (17.531, 18.657)	18	19	5.200	.898	.923	6.036^ns^
SDS	33	[0, 33]^p^ [11, 31]^e^	21.282 (20.826, 21.738)	21	22	4.208	.722	.820	0.639^ns^

*NI* number of items. [Min, Max] = range or interval between minimum and maximum value: p = potential range, e = empirical or observed range. M = arithmetic mean (95% confidence interval). *Mdn* median, *Mo* Mode, α = Cronbach’s alpha based on standardized items. Ordinal α = ordinal coefficient alpha. *PA* = Pearson-Agostino omnibus test for normality and levels of significance for one-tailed test using a chi-square distribution with two degrees of freedom: non-significant (ns) *p* > .05 * *p* ≤ .05, ** *p* ≤ .01, *** *p* ≤ .001. Variables: Age = years of age, *BDI* Beck Depression Inventory-II, *BAI* Beck Anxiety Inventory, *PSS* Parental Stress Scale, *PWS* Psychological Well-being Scale, SDS = Marlowe-Crowne Social Desirability Scale. Source: Prepared by the authors

The correlations between the total score of the RESI-M and its five factors and psychological well-being were significant and positive. The strength of the association with the total score and strength and self-confidence was high, the strength of the association with social competence and family support was medium, and the association with the factors of social support and structure was low (Table 4).

The correlations between the total score of the RESI-M and its factors and depression were significant and negative. The strength of the association with the total score and the factors of strength/self-confidence, social competence and family support was medium, and the association with the factors of social support and structure was low. As was the case for the correlations between parental stress and resilience, the strength of the association with social competence was low (Table 4).

The RESI-M total score and four of its five factors were also correlated negatively with anxiety with a low strength of association. The structure factor was independent of anxiety. In contrast, the RESI-M total score and four of its five factors were independent of social desirability; only the family support factor showed a low and positive correlation with resilience and a shared variance of 1.3% (Table 4).

## Discussion

The first stated objective was to calculate the internal consistency of the total score of the RESI-M and its factors. According to the expectations, very high overall internal consistency was obtained [12, 15–17]. The five factors showed internal consistency values from very high to acceptable, as in other Mexican studies [12, 15, 16]. Among the family caregivers of children with cancer in the present study, no factor had low internal consistency, including the structure factor, which had low internal consistency in another study conducted in Mexico among healthy adults [17].

The second objective was to contrast a five-factor model and examine convergent and discriminant validity in the factors. In agreement with the expectations [12, 15–17], the model showed a close fit to the data and was highly parsimonious. The factors of family support and social support met the criteria for convergent and discriminant validity. The three other factors showed adequate convergent validity because their AVEs were higher than .40 and their composite reliability was higher than .70. The criteria for discriminant validity were fulfilled for five factors, except for the distinction between social competence and structure. The structure factor showed the weakest convergent and discriminant validity properties, in line with our expectations [12, 15–17].

The third objective was to describe the distribution of scores in the RESI-M and its five factors. In line with the expectation of assessing a personality trait [20], the distributions of the total score and the social competence factor followed a normal distribution. The factors of strength/self-confidence and structure had a unique modal value, their measures of central tendency were very close, and both showed symmetry in their tails, with a slightly flattened form in the first and a slightly peaked form in the second. Therefore, their distributions approached normal. The distributions of family support and social support showed negative asymmetry (with a mass of the distribution more concentrated on the right of the arithmetic mean) and had a more acute peak around the arithmetic mean and fatter tails, moving away from a bell-shaped curve. Greater normality was found in the present sample than in previous studies [16, 17]. The distributional characteristics of the support factors could be attributed to the cultural aspects of familism and collectivism [39]. Most of the participants reported receiving considerable support from family and friends.

The fourth objective was to compare the means between the factors. According to the expectations [16], the means of family support, social support and strength/self-confidence had the highest values (without significant differences among the three means). The means of social competence and structure had the lowest values (without significant differences between the two means). There were significant differences between the means of the two groups.

To assess the level of resilience among family caregivers of children with cancer in this study, we can divide the continuous range of scores in the RESI-M and its five factors (1 to 4) into four intervals of constant amplitude ([maximum value-minimum value]/number of values = [4-1]/4 = 0.75) in correspondence with the four ordinal values of the response to the items. In this way, response labels to the items can be used to interpret the scores and measures of central tendency: 1 to 1.749 → 1 = “strongly disagree”, 1.75 to 2.49 → 2 = “disagree”, 2.5 to 3.249 → 3 = “agree”, 3.25 to 4 → 4 = “totally agree”. Following this interpretive approach, the measures of central tendency (means, medians and modes) of the family support factor, in the interval between 3.25 and 4, corresponded to “totally agree” (4), and the measures of the central tendency of the total score of the RESI-M and its four remaining factors, in the interval between 2.5 and 3.249, corresponded to “agree” (3). Therefore, the participants reported a high level of resilience.

The arithmetic mean of the total RESI-M score of the present study, M = 132.715, 95% CI (130.967, 134.463) in a range of 43 to 172, was statistically equivalent, t(774) = − 0.514, *p* = .607 assuming equality of variances through Fisher’s test: F(445, 329) = 1.076, *p* = .240, to the one reported by Toledano-Toledano et al. [15] among 446 Mexican adults with children with chronic diseases, M = 133.330, 95% IC (131.772, 134.888). Nevertheless, the mean of the present study was significantly lower, t(448) = − 13.019, *p* < .001, assuming equality of variance through Fisher’s test, F(119, 329) = 1.017, *p* = .446, than the one reported by Miaja and Moral [16] among 120 Mexican women with cancer, M = 155.167, 95% CI (152.225, 158.109). Therefore, the level of resilience in women with cancer is greater than that in family caregivers of children with cancer. Two other studies in Mexico [12, 17] did not report the arithmetic means of the total RESI-M score and its factors, so we cannot make comparisons.

The fifth objective was to evaluate the relationship with the sociodemographic variables. We expected that the level of resilience would be greater in persons with higher educational levels, older age and female sex [12, 21–23]. This expectation was supported in relation to educational level; in accordance with previous studies, the strength of the association was low [12, 21, 22]. The relationship with age was very low: the greater the age, the greater the level of structure, according to our expectation [12, 21–23]. Older adults have more rules and routines that make it easier for them to maintain organization and order in their lives. However, sex was independent. A similar result was previously reported [15, 17, 22–24, 40]. Although Palomar and Gomez [12] reported greater resilience in women than in men, Fuentes et al. [41] reported more resilience in male adolescents using a resilience questionnaire developed by the authors. In another study published in 2013, the authors reported greater resilience in women in different age groups (children, adolescents, adults from 18 to 30 years old and adults from 31 to 59) [22]. These contradictory results indicate that the effect of sex is spurious and ultimately independent.

The sixth objective was to test the construct validity with respect to psychological well-being (convergent validity) and depression, anxiety and parental stress (divergent validity). Because this study had a transversal design, this construct validity is concurrent validity [42]. We expected a positive correlation with psychological well-being [14] and a negative correlation with depression, anxiety and parental stress [18]. The expectations were fulfilled: resilience, which is a positive personality trait, was more closely related to positive emotions (well-being) than to negative emotions (depression, stress and anxiety), which provides evidence of construct validity for the RESI-M.

The RESI-M total score was independent of social desirability, and only the family support factor had a low correlation. Therefore, it is not necessary to control this variable for bias when the RESI-M is used to assess resilience. The association of social desirability with family support may be related to the aspect of self-deception rather than the aspect of impression management. This type of support is highly valued in Mexico [39]. This affirmation is formulated as a conjecture that could be tested using another scale to measure social desirability that differentiates these two factors, such as the Balanced Inventory of Desirable Responding [43].

The first limitation of this study was the use of nonprobabilistic sampling. Therefore, the inferences are limited to the Hospital Infantil de México Federico Gómez National Institute of Health. However, it should be noted that the case rate was large, covering more than 90% of the incidence of cancer cases per year in this hospital. The second limitation was an ex post facto design; thus, the data do not allow causal inferences. The third limitation was that resilience was assessed through a single self-report scale. Consequently, the conclusions are restricted to this measurement instrument.

## Conclusions

In a sample of 330 family caregivers of children with cancer from the Hospital Infantil de México Federico Gómez National Institute of Health, the overall internal consistency of the RESI-M is very high, and the reliability values of its five factors range from very high to high. The five-factor model is validated, and its five factors show convergent and discriminant validity. The distribution of the total score follows a normal distribution, as does the distribution of the social competence factor. The distributions of the confidence/self-confidence and structure factors are approximately normal, but the distributions of the two support factors are negatively asymmetric and pointed. Therefore, the scale can be scaled using T scores (50 + 20 * standardized score). The level of resilience is high. The average level is higher for the factors of family and social support and strength/self-confidence than for the factors of social competence and structure. A higher level of resilience in the aspects of social competence and family and social support is associated with a higher level of education, and a higher level of structure in daily life is associated with older age. However, sex is independent of the scale. The RESI-M is associated with higher levels of psychological well-being and lower levels of depression, parental stress and anxiety. These relationships with the variables of affective state provide evidence of construct validity. The RESI-M is independent of social desirability, so it does not require control for this variable of bias.

The use of the RESI-M is recommended to assess and study resilience among caregivers of children with cancer. We suggest studying the temporal stability of the scores through test-retest correlation and the temporal stability of the factor structure through a multigroup confirmatory factor analysis. We also propose to provide evidence of predictive validity in relation to the health status of caregivers and children.

## Data Availability

The datasets used and analysed during the current study available from the corresponding author on reasonable request.

## Appendix

### Items of the Mexican Measurement Scale of Resilience (RESI-M)

The RESI-M is composed of forty-three positively keyed items. A higher score indicates greater resilience. The ordinal response categories of each item are the following: 1 = strongly disagree, 2 = disagree, 3 = agree, and 4 = totally agree.

1. What has happened to me in the past makes me feel confident in facing new challenges.
2. I know where to look for help.
3. I am a strong person.
4. I know very well what I want.
5. I have control over my life.
6. I like challenges.
7. I strive to reach my goals.
8. I am proud of my achievements.
9. I know I have skills.
10. Believing in myself helps me overcome difficult moments.
11. I think I will succeed.
12. I know how to achieve my goals.
13. Whatever happens, I will always find a solution.
14. My future looks good.
15. I know that I can solve my personal problems.
16. I am satisfied with myself.
17. I have realistic plans for the future.
18. I trust my decisions.
19. When I am not well, I know that better times will come.
20. I feel comfortable with other people.
21. It is easy for me to establish contact with new people.
22. It is easy for me to make new friends.
23. It is easy for me to think of good topics of conversation.
24. I adapt easily to new situations.
25. It is easy for me to make other people laugh.
26. I enjoy being with other people.
27. I know how to start a conversation.
28. I have a good relationship with my family.
29. I enjoy being with my family.
30. In our family, we are loyal to each other.
31. In our family, we enjoy doing activities together.
32. Even in difficult times, our family has an optimistic attitude toward the future.
33. In our family, we agree in relation to what we consider important in life.
34. I have some friends/relatives who really care about me.
35. I have some friends/relatives who support me.
36. I always have someone who can help me when I need it.
37. I have some friends/relatives who encourage me.
38. I have some friends/relatives who value my skills.
39. Rules and routine make my life easier.
40. I keep my routine even in difficult times.
41. I prefer to plan my activities.
42. I work better when I have goals.
43. I am good at organizing my time.

## Abbreviations

CFA: Confirmatory Factor Analysis
RESI-M: Mexican Measurement Scale of Resilience
